# Lipid Profiles, Telomere Length, and the Risk of Malignant Tumors: A Mendelian Randomization and Mediation Analysis

**DOI:** 10.3390/biomedicines13010013

**Published:** 2024-12-25

**Authors:** Shupeng Liu, Zhengzheng Fu, Hui Liu, Yinghui Wang, Meijuan Zhou, Zhenhua Ding, Zhijun Feng

**Affiliations:** Guangdong Provincial Key Laboratory of Tropical Disease Research, Department of Radiation Medicine, School of Public Health, Southern Medical University, Guangzhou 510515, China; 20220062@smu.edu.cn (S.L.); smufuzz@163.com (Z.F.); lhuidoc@163.com (H.L.); yinghui0618@163.com (Y.W.); lkzmj@smu.edu.cn (M.Z.)

**Keywords:** lipid profiles, telomere length, dyslipidemia, Mendelian randomization, mediation analysis

## Abstract

**Background/Objectives:** The relationship between lipid profiles, telomere length (TL), and cancer risk remains unclear. **Methods:** This study employed two-sample Mendelian randomization (MR) with mediation analysis to investigate their causal relationships, examining lipid profiles as exposure, TL as mediator, and nine cancer types as outcomes. We conducted our analysis using two-stage least squares (2SLS) regression integrated with inverse variance weighted (IVW) methods to address potential endogeneity and strengthen our causal inference. **Results:** we found that unfavorable lipid profiles were causally linked to increased TL (*p* < 0.05). TL showed positive causal associations with lung and hematologic cancers (OR > 1, *p* < 0.05). Direct associations were observed between total and low-density lipoprotein (LDL) cholesterol and gastric cancer (OR < 1, *p* < 0.05), and between remnant cholesterol and colorectal cancer (OR > 1, *p* < 0.05). Mediation analysis revealed TL as a significant mediator in the pathway from lipid profiles to cancer development (*p* < 0.05). No horizontal pleiotropy was detected. **Conclusions:** Our findings suggest that lipid metabolism disorders may influence cancer development through telomere regulation, particularly in lung and hematologic cancers. This emphasizes the importance of lipid management in cancer prevention and treatment, especially for these cancer types.

## 1. Introduction

In recent years, health issues have resulted in considerable socioeconomic costs. Due to the high importance of the management and treatment of cancer, significant efforts have been made to develop new and effective therapeutics for cancer patients [1,2]. The role of dyslipidemia in pathological events and cancer has been a hot topic in biology and public health [3,4,5]. Moreover, researchers have focused on understanding the underlying biological mechanisms involved in the aforementioned pathological events [6,7]. Dyslipidemia is a prominent metabolic disease characterized by abnormal changes in lipids including high-density lipoprotein (HDL) cholesterol, low-density lipoprotein (LDL) cholesterol, total cholesterol (TC), triglyceride (TG), apolipoprotein A-I (APOA1), and apolipoprotein B (APOB). Moreover, remnant cholesterol (RC) has been suggested to be a promising target for pathological events [8]. Hence, it is necessary to provide a better and more comprehensive analysis of lipid profiles and highlight that the effects of dyslipidemia are significantly associated with the initiation and development of various diseases [9,10], particularly atherosclerosis, cardiovascular diseases, pancreatitis, and malignant tumor diseases. Biologically, lipid metabolism has been shown to be associated with different mechanisms, including inflammation [11], oxidative stress [12], cell proliferation [13], and apoptosis [14,15]. Therefore, the dysregulation of lipid metabolism can affect essential biological mechanisms involved in the development of diseases, increasing the risk of human diseases. As a result, controlling the lipid profile is important for treating and managing human diseases.

As an indicator of cellular aging and biological age [16], telomere length (TL), particularly leukocyte telomere length (LTL), is important in the medical field. A reduction in TL can accelerate the aging process, resulting in disorders associated with old age [17]. Hence, telomere dynamics play a significant role in age-associated health conditions. Demographically, dyslipidemia and malignant tumors usually occur in middle-aged and elderly individuals. As a result, it can be concluded that TL, dyslipidemia, and cancer risk are associated with each other, highlighting the crosstalk among aging, lipid metabolism, and cancer development. Notably, an excessively long or short TL is linked to a greater likelihood of developing cancer [18,19]. A lengthy telomere can prevent a “cellular crisis” and preserve the proliferation rate [20,21]. A short telomere increases chromosomal instability, which is responsible for bypassing normal cell division limits and enhancing tumorigenesis [22,23]. As a result, an elongated TL can enhance the viability and propagation of tumor cells and is a potentially important topic for investigation.

Recently, several studies have shown a growing interest in the connection between lipid metabolism dysregulation and cancer development, specifically investigating how lipid metabolism contributes to the occurrence and progression of tumors [24,25,26]. Despite the prevalence of dyslipidemia in the general population, the direct or indirect associations with an increased risk of tumorigenesis have not been completely elucidated. Moreover, there are controversies regarding the impact of numerous specific lipid components on tumorigenesis. Additionally, the relationships among lipid profile, LTL, and the development of malignant tumors remain unclear. Therefore, this study involved the use of a two-sample, two-step Mendelian randomization (MR) analysis utilizing genetic variations as instrumental variables (IVs) to comprehensively investigate the causal relationships among lipid profiles, LTL, and cancer risk, thereby enhancing our understanding of cancer biology.

## 2. Materials and Methods

**Figure 1:**
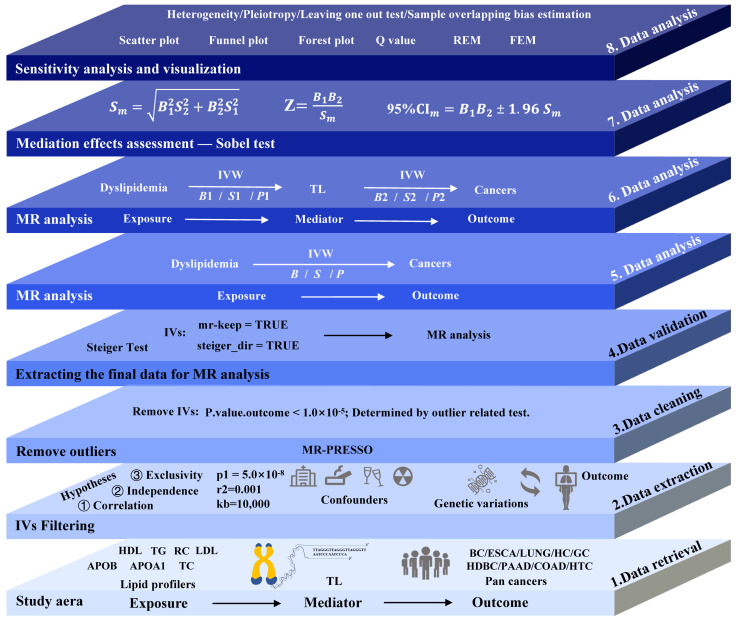
Flowchart of data processing and analytical methods with instrumental variable (IV) selection. Arrows show MR analysis direction. HDL, high-density lipoprotein cholesterol; LDL, low-density lipoprotein cholesterol; TC, total cholesterol; TG, triglyceride; APOA1, apolipoprotein A-I; APOB, apolipoprotein B; RC, remnant cholesterol; BC, brain cancer; LUNG, lung cancer; ESCA, esophageal cancer; GC, gastric cancer; HC, hepatic cancer; HBDC, hepatic bile duct cancer; PAAD, pancreatic cancer; CRC, colorectal cancer; HTC, hematological cancer; TL, telomere length; *B*, estimated causal effect (*B*_1_ and *B*_2_ represent first-stage and second-stage causal effects, respectively); *S*, standard error (*S*_1_ and *S*_2_ denote standard errors for first and second stages); *P*, *p*-value (*P*_1_ and *P*_2_ indicate *p*-values for first and second stages); *Sm*, standard error of mediation; CI, confidence interval; IVW, inverse-variance weighted method; REM, random effect model; FEM, fixed effect model.

This study was based on the guidelines of the STROBE-MR Statement [27]. Figure 1 provides a summary of the different stages of data processing.

### 2.1. Data Source

All the data utilized in this study were sourced from the OpenGWAS database [28]. Datasets related to lipid profiles [29,30,31], including levels of HDL, LDL, TC, TG, APOA1, APOB, and RC, were selected as the exposure data. TL measured in leukocytes served as the mediator [32]. Nine different types of malignant tumors [33], including brain cancer (BC), lung cancer (LUNG), esophageal cancer (ESCA), gastric cancer (GC), hepatic cancer (HC), hepatic bile duct cancer (HBDC), pancreatic cancer (PAAD), colorectal cancer (CRC), and hematological cancer (HTC), were selected as outcomes. Due to the inclusion of both sexes in the study population, sex-specific cancers such as prostate, breast, and ovarian cancer were excluded from the analysis. The data were chosen based on the largest available sample size and the inclusion of mixed-sex European populations. Detailed information about these datasets is presented in Table S1.

### 2.2. Instrumental Variables (IVs) Related to the Exposure and Mediator

Single-nucleotide polymorphisms (SNPs) were identified as IVs for both exposure and mediator using the TwoSampleMR R package (version 0.6.6) [34], with selection parameters (*p* < 5.0 × 10^−8^, r^2^ = 0.001, kb = 10,000) detailed in Figure 1. For MR analysis, the IVs had to meet three core assumptions [35]: strong association with the exposure, independence from confounding factors, and affecting the outcome solely through the exposure (Figure 1). A preliminary examination was conducted for the obtained IVs to ensure the completeness of the sample size and effect allele frequency (EAF) values. The missing sample size was increased by supplementing the original sample with matching datasets from the OpenGWAS database, and the missing EAF values were added using related data from the 1000 Genomes Project for supplementation [36].

### 2.3. Confounding Factor Exclusion Criteria

In this study, the “LDLink” database (https://ldlink.nih.gov/?tab=ldtrait, accessed on 15 October 2024) was used to obtain phenotypic data related to IVs and to identify and eliminate potential confounding IVs [37,38]. Specifically, during the MR analysis with TL as the mediator, SNPs related to TL and telomerase function were excluded to ensure IV independence. For the analysis of cancers affecting different body parts, confounding factors were identified and excluded. These included brain lesions for BC; smoking (including tobacco intake), dust exposure, lung diseases, and malignant diseases for LUNG; esophageal lesions; alcohol consumption for ESCA; Helicobacter pylori infection, atrophic gastritis, other gastric diseases, and alcohol consumption for GC; underlying liver conditions, alcohol consumption for HC and HBDC; pancreatic-related diseases for PAAD; different intestinal diseases for CRC; and work environments involving exposure to radioactive or chemical substances, nontumor diseases of the hematological system, and IVs directly related to blood cells, especially white cells and lymphocytes, for HTC. Additionally, parental history of cancer was considered a potential confounder for all cancer outcomes. Through rigorous selection based on *p*-values and systematic removal of IVs associated with confounders or outcomes, we ensured that the included IVs were independent and adhered to the core assumptions of MR analysis.

### 2.4. Data Extraction and Cleaning

After adjusting for confounding factors, the F-statistics of each IV were calculated, and the IVs with an F greater than 10 were selected [39,40]. Corresponding IVs were extracted from the outcome data pertinent to the current analytical direction. Subsequently, we merged the exposure and outcome data, and IVs associated with outcomes with *p* < 1.0 × 10^−5^ were excluded [41]. The MR-PRESSO outlier test was used to detect and exclude outliers [42]. Additionally, we performed a directionality test to verify the validity of the directional hypotheses [27]. Ultimately, we obtained the whole for inclusion in the final MR analysis [43].

### 2.5. First Steps of the MR Analysis

The first step of the MR analysis was used to assess the overall causal connection between exposure (different types of lipid profiles) and outcome (various cancers). Specifically, MR analysis was conducted using the “TwoSampleMR” R package (version 0.6.8) to obtain odds ratios (ORs) with their corresponding 95% confidence intervals (CIs) [34]. The MR analysis incorporated five distinct methodologies: MR–Egger [44], weighted median (WM) [45], inverse-variance weighting (IVW) [46], simple mode [47], and weighted mode [48]. Causality determination was based on the following rules [27,49]: ① The effect values (*B* values, also known as β values) from the five MR methods must exhibit directional consistency, being either all greater than 0 (positive) or all less than 0 (inverse). ② The statistical significance of the causal relationship is predominantly determined by whether the *P_IVW_* is less than 0.05 [50]. Notably, to uncover the relationship between cancer risk and changes within a specific lipid profile, we performed an exploratory analysis in which the results with *P_IVW_* < 0.05 were displayed. Hence, the Bonferroni correction was not used in this analysis [51]. ③ The criterion for distinguishing between risk factors or protective factors was based on whether the OR was greater than or less than 1.

### 2.6. Second Steps of the MR Analysis

The second step of the MR analysis is primarily focused on intermediary MR analysis, which involves two key directions of analysis (different types of lipids to TL and TL to different types of cancer). For the first direction, the causal relationship of exposure (different types of lipids) with the mediator (TL) was assessed using the two-stage least squares (2SLS) method [52,53]. The estimated *B* values obtained in this direction via the 2SLS method were denoted as *B*_1_ values, with the corresponding standard error (SE) denoted as *S*_1_. In the second direction, five methods of MR analysis were applied to investigate the causal relationship between the mediator (TL) and the outcomes (various types of cancer), and the *B* values obtained with the IVW method were denoted as *B*_2_ values, with the corresponding SEs denoted as *S*_2_. The method of sensitivity analysis in this stage was the same as that used in the first step of the MR analysis.

### 2.7. Assessment of Mediating Effects

The product-of-coefficients method (Sobel test) was used to calculate and evaluate the mediation effects [54,55]. The standard error of mediation (*S_m_*) was initially calculated based on the following formula:

$$S_{m}=\sqrt{B_{1}^{2}S_{2}^{2}+B_{2}^{2}S_{1}^{2}}$$

The Z-statistic is calculated based on *S_m_* as follows:

$$Z=\frac{B_{1}B_{2}}{S_{m}}$$

The Z-statistic analysis revealed a significant mediation effect, indicating that the mediating effect *B_m_* in the current MR analysis was nonzero. These results supported the statistical validity of the mediation pathway. Subsequently, the 95% *CI_m_* for the mediating effects were calculated as follows:

$$95\%CI_{m}=B_{1}B_{2}\pm 1.96S_{m}$$

### 2.8. Sensitivity Analysis

Sensitivity analysis involves analyzing heterogeneity and testing for pleiotropic effects using Cochran’s Q test and MR–Egger methods [42]. The MR–Egger intercept test was used to evaluate horizontal pleiotropy with a significance level of *p* < 0.05 [56]. For IVW, the fixed-effects model was used when there was no heterogeneity, and the random-effects model was used when there was heterogeneity [57].

### 2.9. Assessing Sample Overlap Bias

To evaluate potential sample overlap between exposure and outcome populations, we employed the “mrSampleOverlap” R package [58] to calculate the bias estimation factor (BEF). This factor quantifies the degree of bias in causal estimates that could arise from participant overlap in the exposure and outcome genome-wide association studies (GWAS).

## 3. Results

### 3.1. Direct MR Analysis

In this analysis, a two-step MR approach was employed in which TL was used as the mediator to explore the potential causal relationships between lipid profiles and various cancers across the human body. Following rigorous methodological criteria and procedures, IVs related to various lipids and TL were selected. Detailed information on IVs closely associated with exposure in each direction of the MR analysis is provided in Supplementary Material S1 (Tables S2–S9). After the data cleaning steps, including the exclusion of confounding factors and removal of outliers, final data was obtained to perform MR analysis. Using these data, the feasibility of each analytical direction was explored through “Steiger tests”, and the results (Table S10 in Supplementary Material S1) indicated that the direction of the analysis was precise.

**Figure 2:**
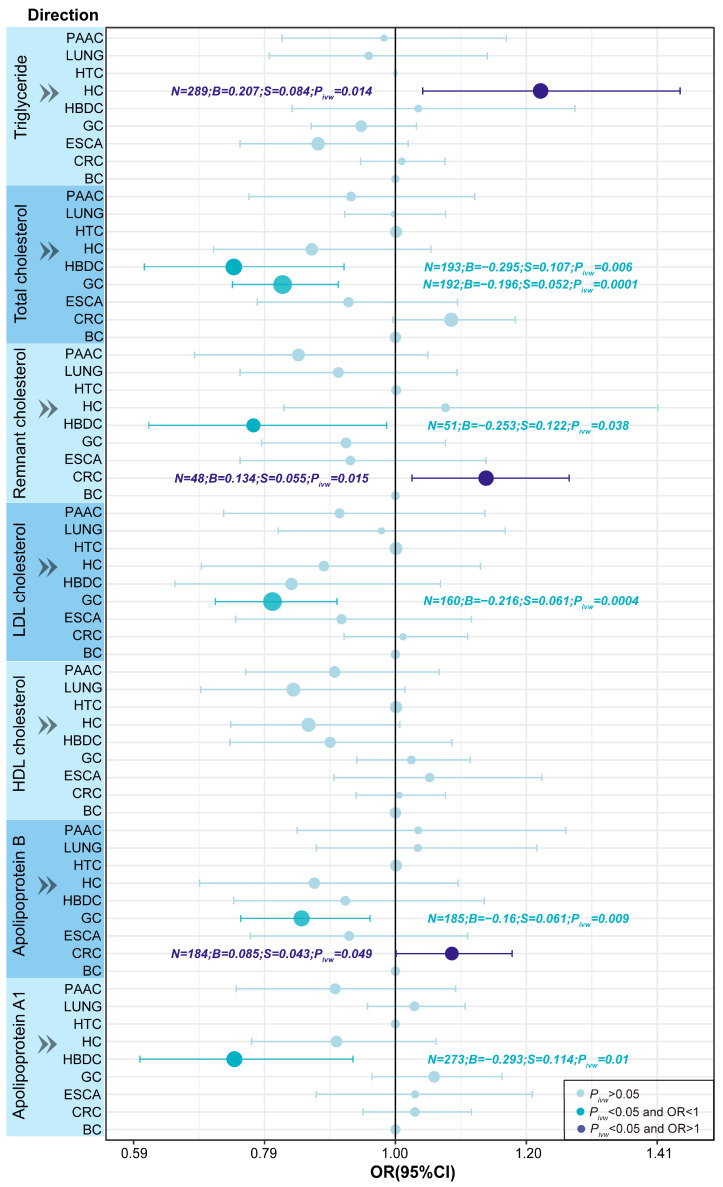
The forest plot illustrates the direct causal effect of the exposures (various types of lipids) on the outcomes (different types of cancer) estimated by the inverse-variance weighted (IVW) method. Dots represent the magnitude of causal effects, and horizontal lines crossing through dots indicate the 95% confidence intervals (CIs) of odds ratios (ORs). Light blue indicates no significant causal relationship between exposure and outcome; blue represents negative causal effects; and dark blue represents positive causal effects between exposure and outcome. BC, brain cancer; LUNG, lung cancer; ESCA, esophageal cancer; GC, gastric cancer; HC, hepatic cancer; HBDC, hepatic bile duct cancer; PAAD, pancreatic cancer; CRC, colorectal cancer; HTC, hematological cancer; TL, telomere length; *N*, the number of instrumental variables used in the corresponding analysis; *B*, effect size; *S*, standard error; *P*, *p*-value.

Notably, to uncover the relationship between cancer risk and changes within a specific lipid profile, we performed an exploratory analysis in which the results with *P_IVW_* < 0.05 were displayed in Figure 2. The results revealed positive causal effects of TG on HC (OR = 1.229, *P_IVW_* = 0.014), RC on CRC (OR = 1.143, *P_IVW_* = 0.015), and APOB on CRC (OR = 1.089, *P_IVW_* = 0.049). However, there was an inverse causal relationship between TC and HBDC (OR = 0.745, *P_IVW_* = 0.006), between TG and GC (OR = 0.822, *P_IVW_* = 0.0001), between LDL and GC (OR = 0.806, *P_IVW_* = 0.004), between APOB and GC (OR = 0.852, *P_IVW_* = 0.009), and between APOA1 and HBDC (OR = 0.746, *P_IVW_* = 0.01). The results obtained from the five MR analysis methods for each analytical direction are provided in Supplementary Materials S1 (Table S11). These findings suggest a complex interplay between lipid profiles and cancer risk. From an exploratory viewpoint, the relationship between lipid profiles and malignant tumors warrants further attention.

### 3.2. Mediation Analysis

This mediation analysis primarily focused on exploring the potential link between various lipid profiles and TL. Judging based on whether the *p*-value was less than 0.05, the results (Table 1) revealed significant positive associations of APOB (*p* = 0.0001), LDL (*p* = 0.0007), TG (*p* = 0.001), TC (*p* = 0.003), and RC (*p* < 0.0001) with TL. Specifically, the R2 values for these lipid profiles were 11.9%, 11%, 6%, 8.7%, and 69.3%, respectively (Table 1). This implies that the potential connection between RC and TL is particularly noteworthy. In contrast, APOA1 and HDL did not significantly impact TL, implying a lack of a significant correlation between these lipids and TL. Overall, these findings reveal a potentially significant positive association between specific lipids, particularly APOB, LDL, TG, TC, and RC, and an increase in TL. These results have important implications for understanding how lipid metabolism contributes to the cellular aging process and the maintenance of TL.

**Figure 3:**
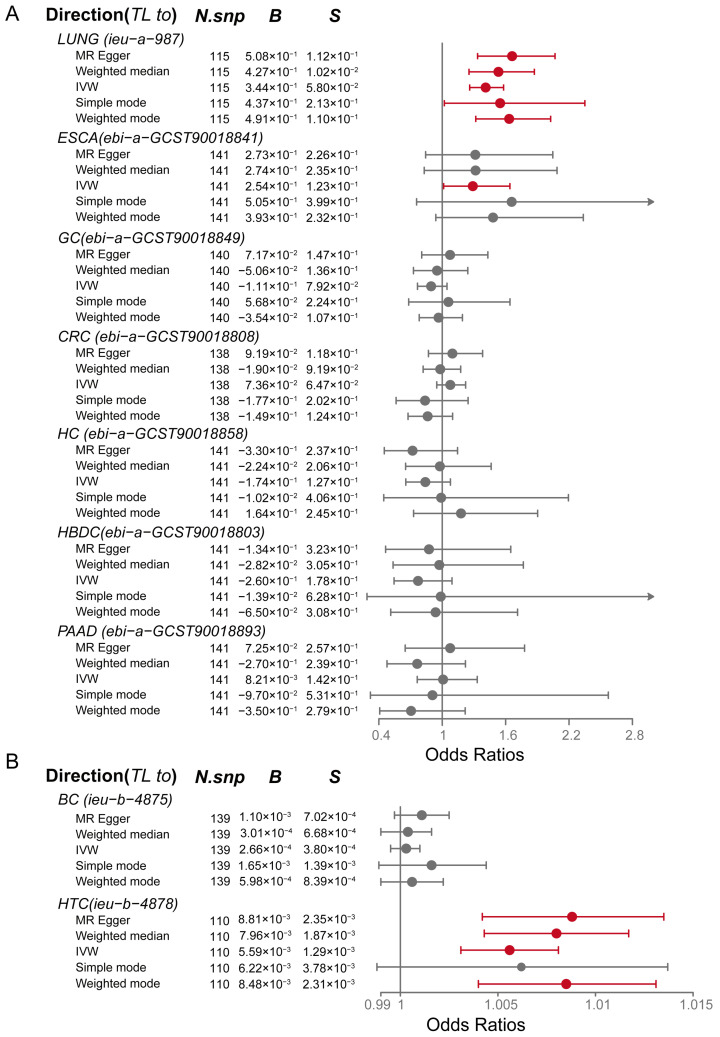
Forest plot shows the results of Mendelian randomization (MR) analysis with telomere length (TL) as the exposure and various cancer types as the outcomes. (**A**), MR analysis between TL and cancer types such as LUNG, ESCA, GC, CRC, HC, HBDC, and PAAD. (**B**), MR analysis between TL and cancer types such as BC and HTC. BC, brain cancer; LUNG, lung cancer; ESCA, esophageal cancer; GC, gastric cancer; HC, hepatic cancer; HBDC, hepatic bile duct cancer; PAAD, pancreatic cancer; CRC, colorectal cancer; HTC, hematological cancer; IVW, inverse-variance weighted method; *N*, the number of SNPs included in the MR analysis; *B*, effect size; *S*, standard error. Abbreviations for cancer types are provided at the end of the text.

In the analysis where TL was considered the exposure factor and various types of cancer were considered the outcomes, the results derived from the IVW method were utilized to determine significance. The comprehensive analysis revealed that TL significantly increased the risk for LUNG (Figure 3A, OR = 1.411, *P_IVW_* < 0.0001), ESCA (Figure 3A, OR = 1.29, *P_IVW_* = 0.039), and HTC (Figure 3B, OR = 1.006, *P_IVW_* < 0.0001). This finding indicates a positive correlation between longer TL and the risk of these specific types of tumors. However, no statistically significant correlation was found when exploring the relationship between TL and other types of cancer, such as gastric, liver, or CRC (Figure 3A,B). The detailed results obtained from the five MR analysis methods for each analytical direction are provided in Supplementary Materials S1 (Table S12). These results suggest that TL may play varying roles in the development of different types of cancer.

In summary, significant analytical findings were observed in both directions: from lipid profiles to TL (Table 1) and from TL to cancer risk (Figure 3). Therefore, these findings provide foundational evidence supporting further exploration into the causal effect of lipids on tumor progression, with TL serving as a mediating factor. These two stages of association underscore the potential relationship between lipid metabolism and cellular aging processes in oncogenesis.

### 3.3. Mediation Effects

Using the coefficient product method to analyze mediation effects (Table 2), the study revealed statistically significant mediation effects of TL between lipid profiles and specific cancer risks. Notably, TL mediated the relationships of TG (*B* = 0.007, *p* = 0.004), TC (*B* = 0.0.012, *p* = 0.007), RC (*B* = 0.029, *p* < 0.0001), LDL (B = 0.011, *p* = 0.003), and APOB (B = 0.001, *p* = 0.0009) with LUNG, as well as TG (*B* = 0.0001, *p* = 0.009), TC (*B* = 0.0002, *p* = 0.012), RC (*B* = 0.0005, *p* = 0.0003), LDL (*B* = 0.0002, *p* = 0.007), APOB (*B* = 0.0002, *p* = 0.003), and HTC (Table 2). TL did not mediate the association between these lipids, and ESCA was statistically significant (Table 2). According to these findings, TL plays a significant role in promoting the development of LUNG and HTC in association with dyslipidemia. Such discoveries not only elucidate the intricate biological mechanisms through which dyslipidemia influences malignant tumor development but also provide important insights for devising targeted prevention and treatment strategies.

### 3.4. Sensitivity Analysis

The results of the sensitivity analysis indicated no significant horizontal pleiotropy in analytically meaningful directions, such as the associations between TL and LUNG, between TL and HTC, or between various lipids and TL (Table S13 in Supplementary Material S1). Despite some analyses showing significant heterogeneity (Table S13 in Supplementary Material S1), the MR analysis results remained consistent when reassessed using a random-effects model (Table S14 in Supplementary Material S1). Scatter (Figure S1 in Supplementary Material S2) and funnel plots (Figure S2 in Supplementary Material S2) of the sensitivity analysis results are also provided, respectively, to aid in a clearer understanding of the data distribution and potential bias.

Additionally, the current study evaluated the potential impact of sample overlap on MR analysis results, and the results revealed that as the sample replication rate increased, the bias in the MR analysis results increased, with some degree of impact on type I errors (Figure S3 in Supplementary Material S2). However, it is noteworthy that this increase in bias and error largely remained within a relatively small range overall. This result suggested that although sample replication has some influence on MR analysis results, the results do not generally deviate significantly from the true values, especially when the sample replication rate is not high.

## 4. Discussion

Using a two-step, two-sample MR analysis, we examined the causal relationships between lipid profiles and various malignant tumors, with particular emphasis on TL as a mediating factor. First, in the two-sample MR analysis examining the total effects between lipid profiles and various cancers, we identified several significant causal relationships. Positive causal effects were observed between TG and HTC, RC, APOB, and CRC. Conversely, negative causal effects were found between TC and both HBDC and GC, between RC and HBDC, between LDL, APOB, and GC, and between APOA1 and HBDC. Second, when examining TL as a mediator, our analysis revealed positive causal relationships between lipid profiles (including LDL, APOB, TC, TG, and RC) and TL, as well as between TL and three cancer types: LUNG, ESCA, and HTC. Subsequently, using the coefficient product method, we evaluated the mediating effects of TL on the relationships between lipid profiles and these three cancers. The results demonstrated significant positive effects through TL for LUNG and HTC, while the mediating effects for ESCA did not reach statistical significance. Sensitivity analyses further validated the robustness and reliability of our findings, lending additional support to the identified causal relationships. These findings provide valuable insights into the complex interplay between metabolic characteristics, aging, and malignant diseases. From a metabolic perspective, our results demonstrate that lipid profiles, as crucial indicators of metabolic health, can significantly influence cancer development through both direct and indirect pathways. This highlights the potential importance of lipid management in cancer prevention strategies. The identification of TL as a significant mediator underscores the fundamental role of aging and genomic stability in carcinogenesis. TL appears to be a key mechanism through which metabolic alterations may influence cancer susceptibility. This finding bridges the gap between metabolic dysregulation and aging processes in cancer development. Regarding malignant diseases, our findings reveal cancer-specific patterns in how metabolic factors, and aging interact to influence cancer risk. The varied relationships observed across different cancer types suggest that the mechanisms linking metabolism to cancer through telomere regulation may be tissue-specific. This understanding could potentially inform more targeted approaches to cancer prevention and intervention strategies, particularly in cancers shown to be influenced by lipid-mediated telomere alterations. These pieces of evidence contribute to our understanding of how modifiable metabolic factors might influence cancer risk through cellular aging pathways, suggesting potential opportunities for cancer prevention through metabolic health management and interventions targeting telomere biology.

This study has significant clinical implications across several key areas. Firstly, our findings have direct relevance for cancer risk management, particularly in LUNG and HTC cases. For patients with these cancers who also present with hyperlipidemia, the condition may be exacerbated through TL-mediated mechanisms. Moreover, in high-risk populations for LUNG or HTC, monitoring both lipid profiles and TL could serve as an effective early warning system. These insights suggest the importance of incorporating these indicators into routine clinical assessment and treatment protocols. Secondly, our direct causal analysis revealed diverse relationships between lipids and specific cancer types. The protective associations were evident in multiple relationships, as TC, APOB, and LDL showed inverse causal relationships with GC, while TC, RC, and APOA1 demonstrated potential protective effects against HBDC. In terms of risk factors, TG levels were associated with increased risk of HC, while RC and APOB levels were identified as risk factors for CRC. Notably, these relationships did not show significant mediating effects through TL, suggesting alternative mechanistic pathways. Finally, this study makes a novel contribution to the field by elucidating TL’s role as a mediator between lipid profiles and cancer risk. The varying patterns observed across different cancer types highlight the complexity and diversity of lipid metabolism in cancer development, suggesting that underlying mechanisms may be cancer-specific. These findings open new avenues for research and provide fresh perspectives on the intricate relationships between metabolic factors and cancer development.

The integration of results from five different MR methods provided robust and comprehensive evidence [59,60], enhancing the reliability of our causal inferences. The integration of these methods ensured a more holistic and reliable understanding of the causal relationships, especially in scenarios where standard MR analysis might be prone to bias due to pleiotropy or other violations of MR assumptions [61]. This observation is similar to the practical situations encountered in this study. The association between lipid abnormalities and cancer is not limited to a single biological pathway involving TL but rather encompasses a broader range of biological mechanisms that were not fully explored in this research [62,63,64]. For instance, our analysis revealed inverse causal relationships between TC/LDL levels and gastric cancer risk, a finding that warrants careful consideration. While this relationship persisted across our MR analyses, and sensitivity tests supported its robustness, it presents an intriguing contrast to previous epidemiological findings [65]. This apparent contradiction may reflect the complex biological mechanisms underlying lipid metabolism in GC development, highlighting the critical need to consider the interplay between genetic predisposition and environmental factors in understanding how lipid metabolism influences gastric cancer pathogenesis.

For the relationship between lipid profiles and CRC, our evidence indicated that elevated levels of RC and APOB might increase CRC risk. These findings align with existing biological evidence suggesting that aberrant lipid metabolism plays a crucial role in colorectal carcinogenesis. Specifically, remnant cholesterol particles can promote inflammation and oxidative stress in the colorectal tissue microenvironment, while elevated APOB levels may reflect broader metabolic dysregulation that creates conditions favorable for tumor development. Given the high incidence and mortality rates of CRC worldwide [66], particularly in developed regions where dietary patterns often lead to adverse lipid profiles, these findings have substantial clinical implications. Clinicians should consider incorporating lipid profile monitoring, especially RC and APOB levels, into CRC risk assessment strategies. Moreover, these results suggest that lipid-lowering interventions might potentially serve as part of comprehensive CRC prevention strategies, although this would require further investigation through clinical trials. The identification of these specific lipid markers also provides new insights for precision medicine approaches in CRC prevention and early intervention.

**Figure 4:**
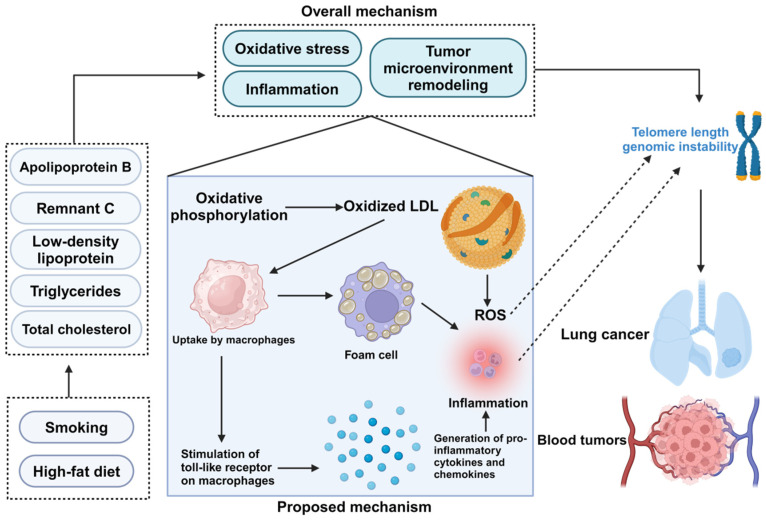
Proposed mechanism connecting lipid components to cancer risk through telomere length alteration.

The literature indicates that lipids may play a role in chronic inflammation and the promotion of oxidative stress within cells [67]. These factors have been linked to the modulation of TL [68] and the progression of cancer [6]. Based on these observations, it is further inferred that lipid profiles may affect the formation and progression of cancers through multiple parallel pathways, which also impact TL on the body. These mechanisms may involve metabolic dysregulation [69], immune modulation [70], and changes in hormone levels [71,72], among other processes (Figure 4). In summary, our findings demonstrate that telomere length serves as a critical mediating factor between lipid profiles and cancer risk, providing mechanistic insight into how metabolic imbalances can disrupt cellular homeostasis and genomic stability, ultimately contributing to increased cancer susceptibility. This relationship underscores the fundamental connection between cellular energy metabolism and the maintenance of genomic integrity in cancer development. In the 2SLS analysis in this work, RC was used as an independent variable, and the R2 value for the relationship between RC and TL reached 0.69. This suggests that RC could be a strong causal explanatory variable for changes in TL and explain 69% of the variability in TL. Similarly, it was observed that the explanatory power of APOB, LDL, TG, and TC on TL is 12% (*R*^2^ = 0.12), 11% (*R*^2^ = 0.11), 6% (*R*^2^ = 0.06), and 9% (*R*^2^ = 0.09), respectively. Although these percentages are lower than those of RC, their statistical significance still indicates that these lipid components contribute to the increase in TL. Similarly, clinical data from an Asian cohort confirmed the elongating effect of persistent hyperlipidemia on TL [73]. Despite the fact that lipid profiles may not be the major influencing factors, this evidence can give us more confidence about the impact of lipid profiles on TL. This further emphasizes the importance of considering lipid components in research on TL variations, particularly when exploring their potential roles in health and disease processes. Given the evidence that elongation of TL increases the risk of LUNG and HTC, monitoring lipid status and overall metabolic status in cancer patients remains an important task in the treatment and management of cancer. By delving into the complex interactions between lipid profiles and cancer risk, future medical research and clinical practice are expected to more effectively prevent and treat these diseases, thereby improving patient survival and quality of life, which is highly important in the field of public health.

In this study, the coefficient product method was utilized to evaluate the mediating effects, which has unique advantages in accurately quantifying the strength of the intermediate variable (such as TL) in causal pathways [74]. In comparison to traditional statistical methods, the coefficient product method can provide a more direct and precise assessment of the size and direction of mediation effects, providing a clearer perspective for understanding complex causal relationships [75]. This study effectively utilized the randomization advantage in the MR design to explore the causal relationships between commonly measured lipid profiles, TL, and cancer risk in clinical settings. This study provides crucial information for the monitoring and management of lipid levels in cancer patients, and also potential early warning biomarkers for high-risk groups of lung and hematologic cancers. Moreover, in our analysis, we did not use the multivariable MR analysis method. This decision is based on the following reasons: multivariable MR analysis typically includes only IVs that relate to specific lipid profiles. However, in the physiological condition of the human body, various lipid components do not exist independently; they coexist and are interwoven. Each lipid has a certain level of expression in the body, and in cases of lipid abnormalities, changes in multiple lipids are usually involved. This implies a continuous interaction among various lipids. Therefore, if the multivariable MR analysis is conducted, it might overlook the complex physiological interactions among these lipids. Such an analysis could lead to neglecting the interaction among different types of lipids, thereby affecting the reliability of the results. Our decision is aimed at ensuring that the results of this study more comprehensively reflect the true nature of physiological processes. Finally, rigorous selection of IVs and exclusion of confounding factors were employed, with no evidence of pleiotropy or violation of MR assumptions found, ensuring the reliability of the study.

Several important limitations of this study should be acknowledged. First, our study primarily relied on GWAS data from European populations, which substantially limits the generalizability of our findings to other ethnic groups. This limitation is particularly noteworthy given that both telomere dynamics and lipid metabolism patterns can vary significantly across different racial and ethnic backgrounds. Future research should address this limitation through the following approaches: (1) conducting similar MR analyses using GWAS data from diverse populations, particularly Asian and African cohorts, to validate these causal relationships across different ethnic groups; (2) performing trans-ethnic meta-analyses to identify both shared and population-specific genetic effects on lipid profiles, telomere length, and cancer risk; and (3) establishing large-scale biobanks and genetic consortia that better represent global populations. These efforts would not only validate our findings but also potentially reveal population-specific mechanisms in the lipid-telomere-cancer relationship that could inform more targeted prevention strategies. Secondly, while our study demonstrated that TL mediates the causal relationship between lipid profiles and certain cancers (LUNG or HTC), the precise molecular mechanisms underlying these associations remain to be fully elucidated. Thirdly, a crucial limitation stems from the use of summary-level data, which precluded access to individual-level information such as age—a particularly important factor given that telomere length naturally decreases with age and varies significantly among individuals. This limitation is especially relevant when considering TL as a mediator, as we cannot account for age-related variations in telomere dynamics or assess how age might modify the observed relationships. Furthermore, the summary-level nature of our data meant that other important individual characteristics, including sex, cancer subtypes, and comorbidities, could not be incorporated into our analyses, preventing more nuanced subgroup investigations that might reveal important effect modifications or population-specific associations.

## 5. Conclusions

Through two-sample, two-step MR analysis, we identified significant causal relationships between lipid profiles, TL, and cancer risk. Initial analysis revealed positive causal associations between specific lipid components (TG, RC, LDL, APOB, and TC) and TL, while subsequent analysis demonstrated that increased TL elevates the risk of particular cancers. Mediation analysis further established the TG/RC/LDL/APOB-TL-LUNG/HTC causal axis, highlighting how lipid levels may influence lung and hematological cancer development through TL regulation. From a mechanistic perspective, these findings reveal a complex biological pathway: lipid metabolism disorders appear to disrupt cellular homeostasis by affecting telomere maintenance, while abnormally elongated telomeres may confer unlimited replication potential to cancer cells, consistent with our observation of increased cancer risk associated with telomere lengthening. The complementary findings from both analytical steps provide strong evidence for the role of telomere biology in mediating the relationship between lipid metabolism and cancer development, while also offering empirical support for the clinical importance of lipid management in cancer patients.

## Figures and Tables

**Table 1:** Assessment of the causal link between lipid profiles and telomere length (TL).

Coefficients	*B*	*S*	t	*P*	*R* ^2^
***APOA1 (ieu-b-107) to TL***					
Intercept	2.37 × 10^−4^	3.08 × 10^−4^	0.77	4.43 × 10^−1^	2.55 × 10^−4^
Predicted	−1.76 × 10^−3^	7.82 × 10^−3^	−0.23	8.23 × 10^−1^	
***APOB (ieu-b-108) to TL***					
Intercept	−2.59 × 10^−4^	4.03 × 10^−4^	−0.64	5.22 × 10^−1^	1.19 × 10^−1^
Predicted *	2.97 × 10^−2^	7.44 × 10^−3^	3.99	1.17 × 10^−4^	
***HDL (ieu-b-109) to TL***					
Intercept	2.17 × 10^−4^	2.97 × 10^−4^	0.73	4.64 × 10^−1^	4.61 × 10^−4^
Predicted	2.35 × 10^−3^	7.12 × 10^−3^	0.33	7.42 × 10^−1^	
***LDL (ieu-b-110) to TL***					
Intercept	2.36 × 10^−4^	4.00 × 10^−4^	0.59	5.57 × 10^−1^	1.10 × 10^−1^
Predicted *	3.32 × 10^−2^	9.53 × 10^−3^	3.48	7.44 × 10^−4^	
***TG (ieu-b-111) to TL***					
Intercept	1.22 × 10^−4^	3.07 × 10^−4^	0.39	6.91 × 10^−1^	6.05 × 10^−2^
Predicted *	2.17 × 10^−2^	6.67 × 10^−3^	3.25	1.40 × 10^−3^	
***TC (ebi-a-GCST90025953) to TL***					
Intercept	1.24 × 10^−4^	3.60 × 10^−4^	0.35	7.31 × 10^−1^	8.76 × 10^−2^
Predicted *	3.45 × 10^−2^	1.13 × 10^−2^	3.05	2.93 × 10^−3^	
***RC (ebi-a-GCST90092943) to TL***					
Intercept	1.86 × 10^−4^	7.56 × 10^−4^	0.25	8.09 × 10^−1^	6.93 × 10^−1^
Predicted *	8.37 × 10^−2^	1.31 × 10^−2^	6.37	5.35 × 10^−6^	

HDL, high-density lipoprotein cholesterol; LDL, low-density lipoprotein cholesterol; TC, total cholesterol; TG, triglyceride; APOA1, apolipoprotein A-I; APOB, apolipoprotein B; RC, remnant cholesterol; TL, telomere length; *B*, estimated coefficient value; *S*, standard error; t, t statistic; *P*, *p*-value; *R*^2^, coefficient of determination. * indicates that the corresponding result of this entry is statistically significant.

**Table 2:** The results of mediation effects in Mendelian randomization (MR) analysis.

Direction	*B_m_*	*S_m_*	Z	*P_m_*
TG → TL →				
LUNG	7.46 × 10^−3^	2.62 × 10^−3^	2.85	4.34 × 10^−3^
HTC	1.21 × 10^−4^	4.66 × 10^−5^	2.60	9.28 × 10^−3^
ESCA	5.51 × 10^−3^	3.22 × 10^−3^	1.71	8.66 × 10^−2^
TC → TL →				
LUNG	1.19 × 10^−2^	4.37 × 10^−3^	2.71	6.64 × 10^−3^
HTC	1.93 × 10^−4^	7.73 × 10^−5^	2.50	1.26 × 10^−2^
ESCA	8.76 × 10^−3^	5.21 × 10^−3^	1.68	9.25 × 10^−2^
RC → TL →				
LUNG	2.88 × 10^−2^	6.62 × 10^−3^	4.35	1.38 × 10^−5^
HTC	4.68 × 10^−4^	1.30 × 10^−4^	3.59	3.35 × 10^−4^
ESCA	2.13 × 10^−2^	1.11 × 10^−2^	1.92	5.45 × 10^−2^
LDL → TL →				
LUNG	1.14 × 10^−2^	3.80 × 10^−3^	3.00	2.67 × 10^−3^
HTC	1.86 × 10^−4^	6.84 × 10^−5^	2.72	6.63 × 10^−3^
ESCA	8.43 × 10^−3^	4.83 × 10^−3^	1.74	8.10 × 10^−2^
APOB → TL →				
LUNG	1.02 × 10^−2^	3.09 × 10^−3^	3.31	9.27 × 10^−4^
HTC	1.66 × 10^−4^	5.65 × 10^−5^	2.94	3.32 × 10^−3^
ESCA	7.54 × 10^−3^	4.19 × 10^−3^	1.80	7.19 × 10^−2^

HDL, high-density lipoprotein cholesterol; LDL, low-density lipoprotein cholesterol; TC, total cholesterol; TG, triglyceride; APOA1, apolipoprotein A-I; APOB, apolipoprotein B; RC, remnant cholesterol; BC, brain cancer; LUNG, lung cancer; ESCA, esophageal cancer; GC, gastric cancer; HC, hepatic cancer; HBDC, hepatic bile duct cancer; PAAD, pancreatic cancer; CRC, colorectal cancer; HTC, hematological cancer; TL, telomere length; *B_m_*, mediation effect size; *S_m_*, standard error for mediation effect; Z, Z statistics; *P_m_*, *p*-value for mediation effect.

## Data Availability

The datasets generated and/or analyzed during this study are available at the OpenGWAS project website (https://gwas.mrcieu.ac.uk/, accessed on 15 September 2024).

## Supplementary Materials

The following supporting information can be downloaded at: https://www.mdpi.com/article/10.3390/biomedicines13010013/s1, Figure S1: Scatter plots for all MR analyses; Figure S2: Funnel plot for all MR analyses; Figure S3: Impact of sample overlap on MR analysis results. Table S1: GWAS datasets for the study; Table S2: IVs and F-Values of APOA1; Table S3: IVs and F-Values of APOB.; Table S4: IVs and F-Values of HDL; Table S5: IVs and F-Values of LDL; Table S6: IVs and F-Values of TG; Table S7: IVs and F-Values of TC; Table S8: IVs and F-Values of RC; Table S9: IVs and F-Values of TL; Table S10: The Steiger test for the direction of MR analysis; Table S11: MR results between lipid and cancers; Table S12: MR results between TL and cancers; Table S13: pleiotropy and heterogeneity results; Table S14: The MR results of random effect model.
