# Supplementary material for: Lipid Profiles, Telomere Length, and the Risk of Malignant Tumors: A Mendelian Randomization and Mediation Analysis
Source: Biomedicines. 2024 Dec 25;13(1):13. doi: 10.3390/biomedicines13010013 (PMC11760878; doi:10.3390/biomedicines13010013)
Supplement: Supplementary file 1 [file biomedicines-13-00013-s001.zip › Table S1 GWAS datasets for the study.pdf]

Table S1 The data sets used in the MR analysis

GWAS ID	Year	Trait	Population	Sex	N_{Sample}	N_{SNP}	Author	Type
**Exposures**								
ieu-b-107	2020	Apolipoprotein A-I	European	Males and Females	393,193	12,321,875	Richardson, Tom	Continuous
ieu-b-108	2020	Apolipoprotein B	European	Males and Females	439,214	12,321,875	Richardson, Tom	Continuous
ieu-b-109	2020	HDL cholesterol	European	Males and Females	403,943	12,321,875	Richardson, Tom	Continuous
ieu-b-110	2020	LDL cholesterol	European	Males and Females	440,546	12,321,875	Richardson, Tom	Continuous
ieu-b-111	2020	Triglycerides	European	Males and Females	441,016	12,321,875	Richardson, Tom	Continuous
ebi-a-GCST90025953	2021	Total cholesterol levels	European	Males and Females	437,878	4,232,052	Barton AR	Continuous
ebi-a-GCST90092943	2022	Remnant cholesterol	European	Males and Females	115,082	11,590,399	Richardson TG	Continuous
**Mediator**								
ieu-b-4879	2021	Telomere length	European	Males and Females	472,174	20,134,421	Codd	Continuous
**Outcomes**								
ieu-a-987	-	Lung cancer	European	Males and Females	85,449	10,439,018	TRICL	Binary
ieu-b-4875	2021	Brain cancer	European	Males and Females	372,622	8,629,116	Burrows	Binary
ebi-a-GCST90018841	2021	Esophageal cancer	European	Males and Females	476,306	24,194,380	Sakaue S	Binary
ebi-a-GCST90018849	2021	Gastric cancer	European	Males and Females	476,116	24,188,662	Sakaue S	Binary
ebi-a-GCST90018808	2021	Colorectal cancer	European	Males and Females	470,002	24,182,361	Sakaue S	Binary
ebi-a-GCST90018858	2021	Hepatic cancer	European	Males and Females	475,638	24,194,938	Sakaue S	Binary
ieu-b-4878	2021	Haemotological cancer	European	Males and Females	376,568	11,562,585	Burrows	Binary
ebi-a-GCST90018803	2021	Hepatic bile duct cancer	European	Males and Females	476,091	24,196,592	Sakaue S	Binary
ebi-a-GCST90018893	2021	Pancreatic cancer	European	Males and Females	476,245	24,195,229	Sakaue S	Binary
