# Supplementary figures and images for: Lipid Profiles, Telomere Length, and the Risk of Malignant Tumors: A Mendelian Randomization and Mediation Analysis
Source: Biomedicines. 2024 Dec 25;13(1):13. doi: 10.3390/biomedicines13010013 (PMC11760878; doi:10.3390/biomedicines13010013)

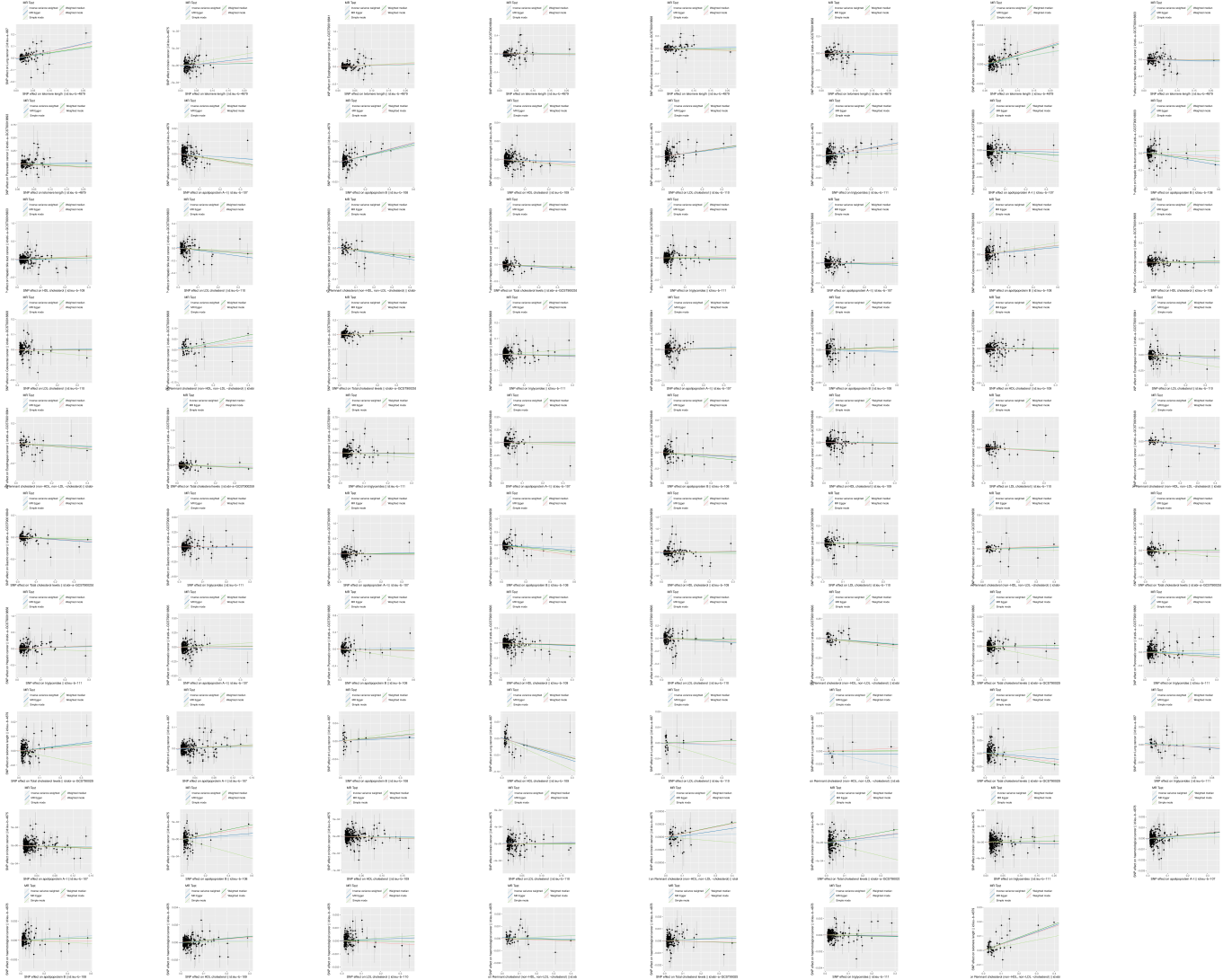

Supplement: Supplementary file 1 [file biomedicines-13-00013-s001.zip › Figure S1 Scatter plots for all MR analysis.pdf]

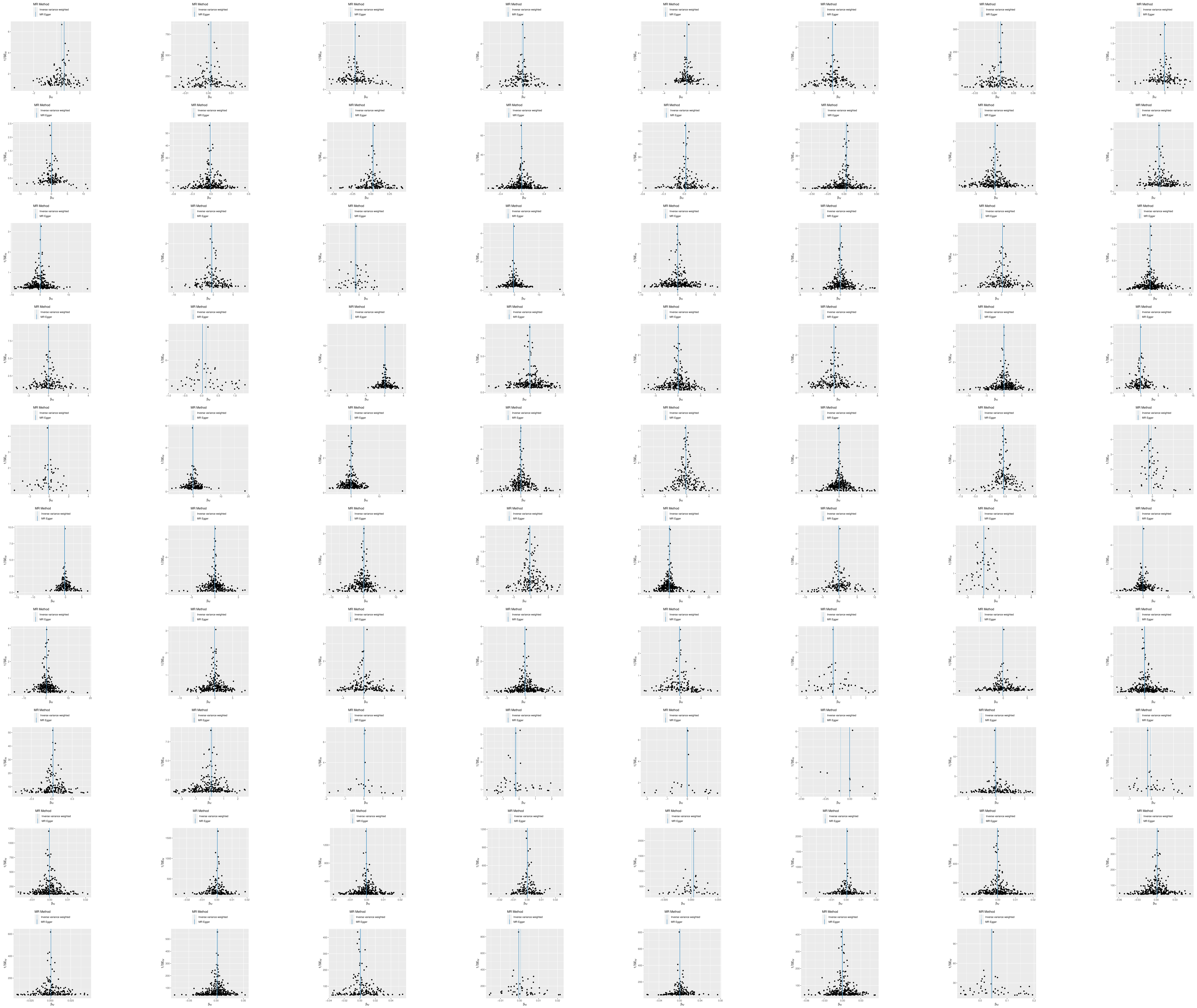

Supplement: Supplementary file 1 [file biomedicines-13-00013-s001.zip › Figure S2 Funnel plot for all MR analysis.pdf]

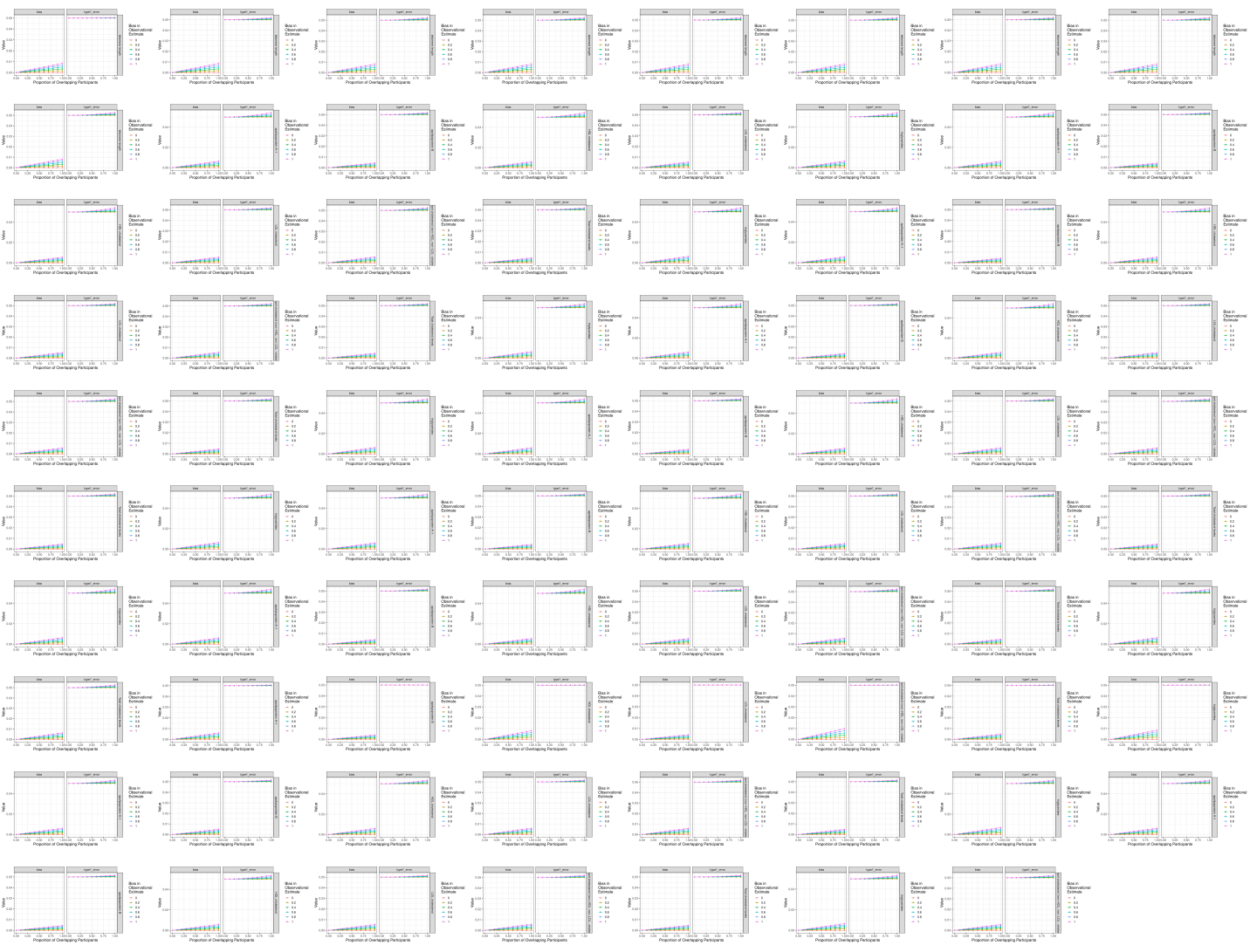

Supplement: Supplementary file 1 [file biomedicines-13-00013-s001.zip › Figure S3 Impact of sample overlap on MR analysis results.pdf]
